# High Refractive Organic–Inorganic Hybrid Films Prepared by Low Water Sol-Gel and UV-Irradiation Processes

**DOI:** 10.3390/nano6030044

**Published:** 2016-03-09

**Authors:** Hsiao-Yuan Ma, Tzong-Liu Wang, Pei-Yu Chang, Chien-Hsin Yang

**Affiliations:** Department of Chemical and Materials Engineering, National University of Kaohsiung, No. 700, Kaohsiung University Road, Kaohsiung 811, Taiwan; 295329@cpc.com.tw (H.-Y.M.); tlwang@nuk.edu.tw (T.-L.W.); m1045614@mail.nuk.edu.tw (P.-Y.C.)

**Keywords:** optical thin film, high refractive index, organic/inorganic hybrid, sol gel method

## Abstract

Organic-inorganic hybrid sols (Ti–O–Si precursor) were first synthesized by the sol-gel method at low addition of water, and were then employed to prepare a highly refractive hybrid optical film. This film was obtained by blending the Ti–O–Si precursor with 2-phenylphenoxyethyl acrylate (OPPEA) to perform photo-polymerization by ultraviolet (UV) irradiation. Results show that the film transparency of poly(Ti–O–Si precursor-co-OPPEA) film is higher than that of a pure poly(Ti–O–Si precursor) film, and that this poly(Ti–O–Si precursor-co-OPPEA) hybrid film exhibits a high transparency of ~93.7% coupled with a high refractive index (*n*) of 1.83 corresponding to a thickness of 2.59 μm.

## 1. Introduction

Flat panel displays, imaging sensors, photonic circuits, and light-emitting diodes, can be improved their performance by employing a transparent high refractive index (RI) coating (≥1.65) to the light-emitting part in the device. The light is more effectively traveled into or out of the device transiting from the high refractive index of the active circuitry to the low index of air, improving its efficiency and image quality. In general, the excellent durability and easy deposition of a spin-coating polymer with the high refractive index and optical clarity of a vacuum-deposited metal oxide coating have been developed. Titanium dioxide and zirconium oxide are common-used coating materials.

An effective strategy is employed to add inorganic components with high molar refractions into polymer structures for improving refractive index and retaining a high Abbe number. For example, an alternating ferrocene unit [1] and a –P=N– backbone introducing in polymers [2,3] has a maximum RI of 1.7. Using these approaches, however, is difficult to obtain a RI higher than 1.8 for a polymer material. An alternative approach of organic–inorganic hybrid materials possesses outstanding physical and chemical properties coming from the nature of organic–inorganic hybrid [4,5]. Some achievements have been reached through the combination of organic and inorganic materials generating transparent hybrid coatings, typically, dispersing a metal oxide sol in a polymer matrix to produce a creamer [6]. On the other hand, a metal oxide precursor is hydrolyzed and condensed *in situ* to obtain highly dispersed metal oxide which it is chemically bonded with a polymer matrix using the sol-gel method [7,8]. Refractive index is generally increased from 1.6 to 1.7 in the resulting film using the above-mentioned approaches where the metal oxide component is present in the matrix prior to coating. It is well-known that titanium oxide has been extensively used in the formation of sol-gel materials for optical applications for its high refractive index [9,10,11,12,13,14,15]. Recently, photonic nanosheets have been demonstrated by using sol-gel derived titanate [16]. Considering the reactivity of the titanium alkoxides, chelating additives or pre-formed titanium oxide nanoparticles were added and then dispersed into an organic polymer matrix in sol-gel polymerizations of alkoxides. Generally, nanoparticle composites would show scattering loss at high loading and are hard to process. We have tried to prepare the hybrid systems which nanoparticles disperse well without precipitation.

Here we report the first methacryloxypropyltrimethoxysilane (MAPTMS) mediated sol-gel polymerization of titanium(IV) isopropoxide (TTIP) at low addition of water, in a one-pot synthesis to obtain the Ti–O–Si precursor. The methacrylate functionality in Ti–O–Si precursor allowed us to mix 2-phenylphenoxyethyl acrylate (OPPEA) and directly photopattern the obtained high quality thin films of organically modified titanium oxide with high refractive index of 1.83 at 633 nm. The high refractive-index metal oxide component forms simultaneously during the curing process of the coating, generating the polymer matrix with dispersed metal oxide-nanoparticle phases. The dispersions of the Ti–O–Si precursors in acrylic resin are stable at room temperature for a minimum of 6 months and can easily use the spin-coating to form a uniform film that are several micro-meter (μm) thick having highly transparent in the wavelength range of 500 to 1000 nm through the UV-induced polymerization. This hybrid coating can be evaluated and used widely for optical applications.

## 2. Results

### 2.1. Sol-Gel Formation

**Figure 1:**
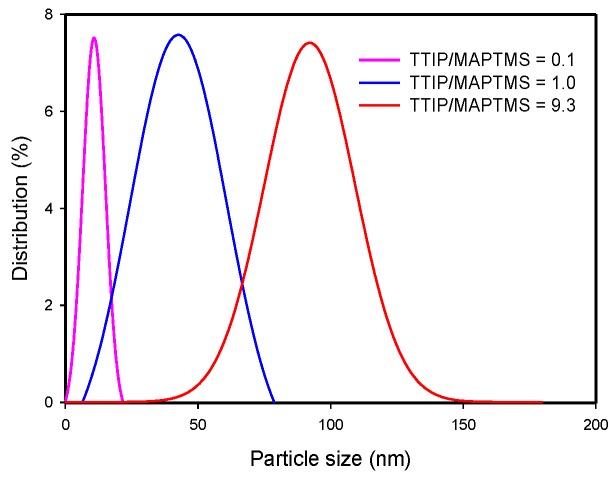
The dynamic light scatter data plot of Ti–O–Si-organic hybrid sols with several titanium(IV) isopropoxide/methacryloxypropyltrimethoxysilane (TTIP/MAPTMS) ratios.

**Figure 2:**
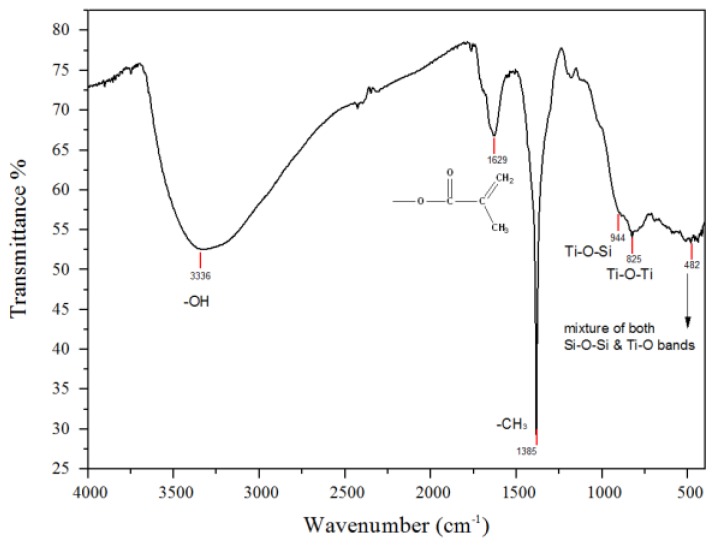
Fourier transform infrared spectra (FTIR) of Ti–O–Si-organic precursor sols.

**Figure 3:**
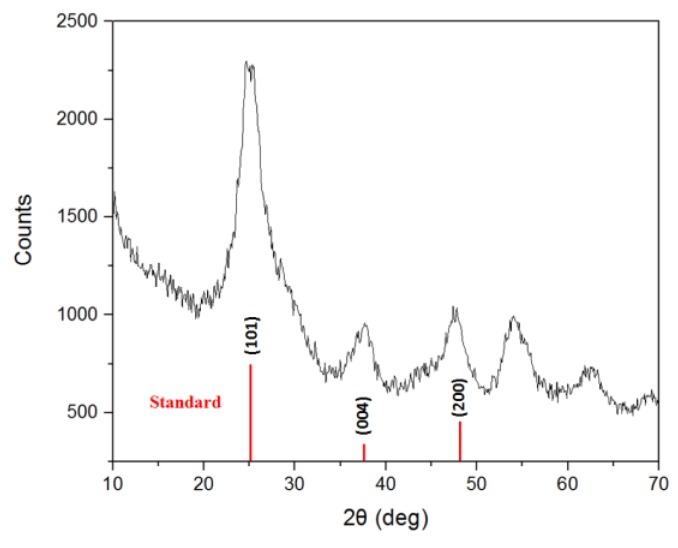
X-ray diffraction (XRD) pattern of Ti–O–Si-organic precursor.

**Figure 4:**
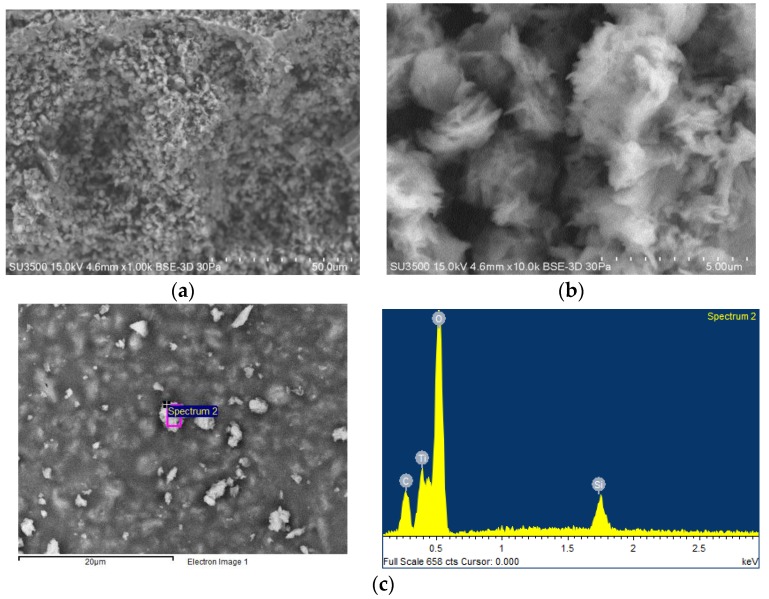
(**a**,**b**) Scanning electron microscopy (SEM) morphology of the Ti–O–Si-organic precursor; (**c**) Energy Dispersive Spectrometer (EDS) elemental analysis.

The average particle size of Ti–O–Si-organic precursors was determined by the size distribution of particles in a typical dynamic light scatter data plot (Figure 1). The average particle size of Ti–O–Si-organic precursors is 93, 43, and 11 nm with a TTIP/MAPTMS ratio of 9.3, 1.0, and 0.1, respectively. Chemical transformations during the synthesis of organic-inorganic hybrid sol were identified using Fourier transform infrared (FTIR), X-ray diffraction (XRD), and light-scattering spectroscopy. The product of the sol-gel process was confirmed using FTIR spectroscopy as shown in Figure 2. The characteristic IR absorption bands of Si–O–Si and Ti–O, Ti–O–Si, and Ti–O–Ti appear at 440 cm^−1^ [17], 940 cm^−1^ [18], and 825 cm^−1^ [19], respectively. The bonding of the acrylate groups on the organic-inorganic hybrid sol can be observed by the presence of the characteristic CH=CH_2_ at 1629 cm^−1^. The characteristic bands of sym. C–H bending and –OH stretching on the organic-inorganic hybrid sol were present at 1385 [19] and 3336 cm^−1^, respectively. Figure 3 shows the X-ray diffraction (XRD) pattern of the resulting organic-inorganic hybrid precursor. According to Joint Committee on Powder Diffraction Standards (JCPDS) data, the peaks at 2θ = 25.2°, 37.6°, 48.1°, 54.1°–55.2°, 62.6°, and 69.0° correspond to the (1 0 1), (0 0 4), (2 0 0), (1 0 5)–(2 1 1), (2 0 4), and (1 1 6) planes of anatase TiO_2_, respectively. In fact, the peaks of 54.1° and 55.2° merge together to form a broad peak of (1 0 5) and (2 1 1) planes. Figure 4 shows the scanning electron microscopy (SEM) morphology of the Ti–O–Si-organic precursor, demonstrating silk-like structures having the elemental profile (weight percent) of C:O:Si:Ti = 7.0:58.5:1.6:32.9.

### 2.2. Organic-Inorganic Hybrid Polymer Films

**Figure 5:**
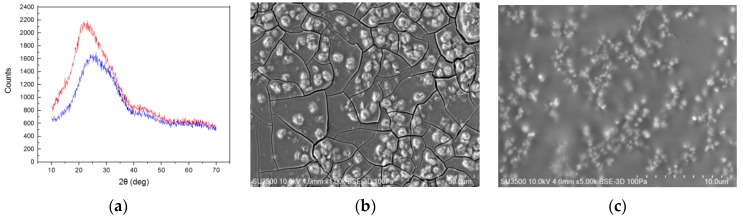
(**a**) XRD patterns of poly(Ti–O–Si precursor) (**–**) and poly(Ti–O–Si precursor-co-OPPEA) hybrid films (**–**). SEM images of (**b**) poly(Ti–O–Si precursor) and (**c**) poly(Ti–O–Si precursor-co-OPPEA) hybrid films. (OPPEA: 2-phenylphenoxyethyl acrylate).

**Figure 6:**
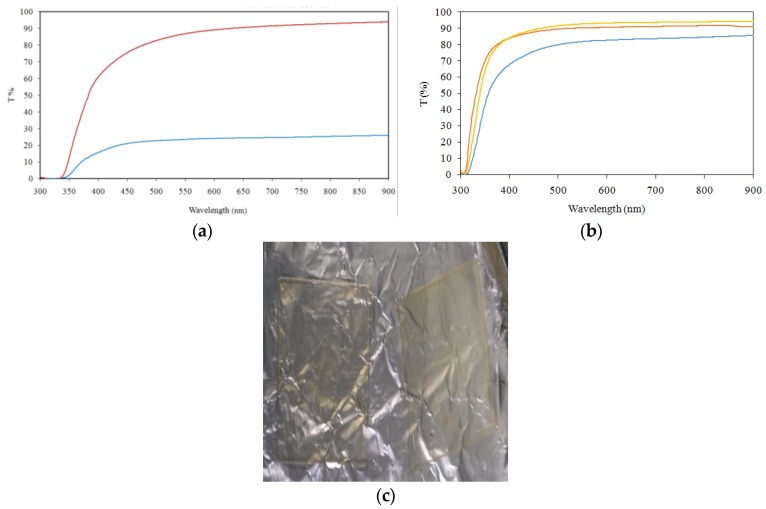
Ultraviolet–visible (UV-vis) spectra of (**a**) poly(Ti–O–Si precursor) (**–**) (6.75 μm) and poly(Ti–O–Si precursor-co-OPPEA) hybrid films (**–**) (6.77 μm) and (**b**) poly(Ti–O–Si precursor-co-OPPEA) hybrid films with different thicknesses (**–**) 2.59, (**–**) 6.77, and (**–**) 11.75 μm. (**c**) Photograph of poly(Ti–O–Si precursor-co-OPPEA) hybrid film (2.59 μm).

**Figure 7:**
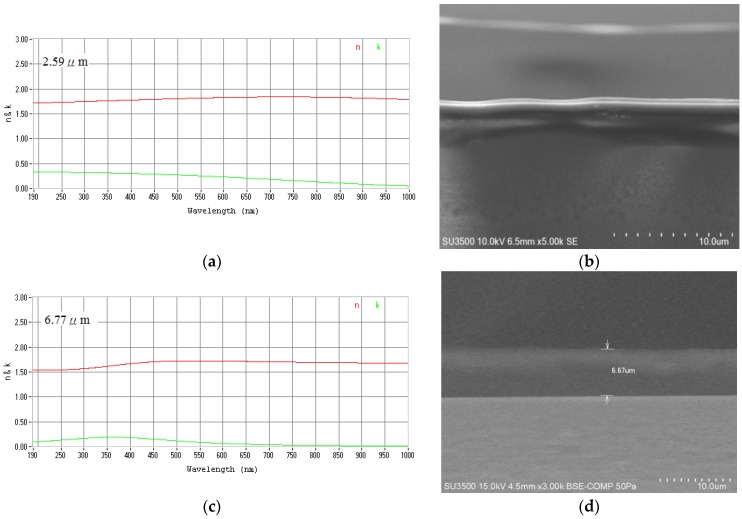
Refractive index (*n*) and extinction coefficient (*k*) of poly(Ti–O–Si precursor-co-OPPEA) hybrid films with thicknesses of (**a**) 2.59 and (**b**) 6.77 μm. The estimated thickness was confirmed by the cross-section view of films in the SEM image.

The XRD patterns of the resulting organic-inorganic hybrid polymer films are shown in Figure 5a. In contrast to Figure 3, these organic-inorganic hybrid polymer films exhibit amorphous structure after photopolymerization of both the Ti–O–Si precursor/OPPEA mixture and pure Ti–O–Si precursor. The crystal planes of anatase TiO_2_ are almost suppressed and only highlight a broad (1 0 1) plane. The peak of the former hybrid shifts to a smaller angle (2θ) of *ca*. 23° relative to the values of 25° and 25.2° for the latter hybrid and the precursor (refer to Figure 3), respectively. Based on Scherrer’s equation, the average crystal lattice on the (1 0 1) plane is in the order: Ti–O–Si precursor (3.0 nm) > poly(Ti–O–Si precursor) (0.63 nm) > poly(Ti–O–Si precursor-co-OPPEA) (0.58 nm). Figure 5b shows a crack film of the poly(Ti–O–Si precursor) hybrid. In contrast, a uniformly compact film of the poly(Ti–O–Si precursor-co-OPPEA) hybrid is shown in Figure 5c. Figure 6a shows the UV-vis spectra of the organic-inorganic hybrid polymer films with a thickness of ~6.8 μm, revealing that poly(Ti–O–Si precursor-co-OPPEA) and poly(Ti–O–Si precursor) hybrid films at 633 nm have a transmittance of 91.1 and 25.2%, respectively. Poly(Ti–O–Si precursor-co-OPPEA) hybrid films at 633 nm have different transmittances of 82.2, 91.1, and 93.7% corresponding to different thicknesses of 11.75, 6.77, 2.59 μm, respectively (Figure 6b). Figure 6c shows the photograph of a 2.59-μm-thick poly(Ti–O–Si precursor-co-OPPEA) hybrid film. The refractive index of poly(Ti–O–Si precursor-co-OPPEA) hybrid films was measured on a N & K analyzer and is shown in Figure 7. The refractive indices increase with decreasing the film thickness in the heterogeneous hybrid films, which are 1.83 and 1.72 at the visible wavelength of 633 nm, corresponding to the film thicknesses of 2.59 and 6.77 μm, respectively. Also, note that the estimated film thickness from the N & K analyzer can be confirmed by the cross-section view of the hybrid films in the SEM image (Figure 7a,b). On the other hand, the light transmittance is only 82.2% in 11.75-μm-thick film, which does not meet the conditions of high light transmittance that is >90% (Figure 6b). In practice, the refractive index of LED chips ranges from 2.5 to 3.5, whereas that of the polymer encapsulation materials is in the range of 1.5–1.6. If the refractive index of the polymer materials were raised to 1.8, the efficiency of LED light emission can be promoted about 40%. Therefore, the development of high-refractive-index encapsulation materials is the most important issue in the LED industry. In this work, since we have first ensured high transmittance (>90%) in a practical application, an optical film can be prepared to give a high refractive index of 1.83 with a film thickness of 2.59 μm.

## 3. Discussion

### 3.1. Sol-Gel Formation

In Figure 1, the particle size of the Ti–O–Si-organic precursors increases with increasing the ratio of TTIP/MAPTMS, because the TTIP molecule has four reactive isopropoxides which readily result in a network structure. More TTIP content in the precursor leads to more networks, enlarging the resulting particle size.

FTIR results are shown in Figure 2. These presented bands indicate that TTIP and MAPTMS have successful hydrolysis and condensation to generate organic-inorganic hybrid sol.

The formation of the anatase phase of TiO_2_ is shown in Figure 3. The theoretical mole ratio of Ti and Si in the reactants is about 9.3:1, leading to the homogeneous condensation of TiO_2_ particles.

Figure 4 shows the SEM morphology of the Ti–O–Si-organic precursor, demonstrating silk-like structures having the elemental profile (weight percent) of C:O:Si:Ti = 7.0:58.5:1.6:32.9. The mole ratio of Ti and Si in the hybrid precursor is about 11.7:1, resulting from the homogeneous condensation of TiO_2_ particles because the reactivity of TTIP is greater than that of MAPTMS. Correspondingly, the high content of TiO_2_ is expected to obtain a high-refractive-index material. In the other samples of TTIP/MAPTMS ratios of 1.0 and 0.1, the content of MAPTMS in the two ratios would lead to a relatively low refractive index in the resulting poly(Ti–O–Si precursor-co-OPPEA) hybrid film. Therefore, these two will not be discussed in the following section.

### 3.2. Organic-Inorganic Hybrid Polymer Films

In Figure 5a of the XRD pattern, these organic-inorganic hybrid polymer films exhibit an amorphous structure through the photopolymerization of both the Ti–O–Si precursor/OPPEA mixture and pure Ti–O–Si precursor. The crystal planes of anatase TiO_2_ are almost suppressed in polymer forms, with a decrease of the average crystal lattice on the (1 0 1) plane. This suggests that the bonding distance of the inorganic Ti–O–Si segments would be suppressed by densitification through the photopolymerization of Ti–O–Si precursor. In general, hybrid inorganic-organic creamer film is a typical example without macrophase separation. However, compositional deviation exists on a microscopic level *ca*. 10 nm as evidenced by small-angle X-ray scattering (SAXS) [20,21]. The SAXS data provide information on any systematic variation in the average electron density of a scattering source. The concentration variation in the creamer films depend on the compatibility of the organic and inorganic components which successively influences the nature of growth and resulting structures of the inorganic phase. A general morphological model was proposed for this microphase behavior consisting of three regimes (phases) which make up the creamer morphology: the organic-rich regimes, organic and inorganic mixed regimes, and the inorganic-rich regimes. The spacing between the inorganic-rich regimes measured using SAXS is represented by the average interdomain spacing d. The separation of a dispersed inorganic-rich phase chemically crosslinked at the interface with an organic-rich matrix phase leads to the generation of an interdomain spacing. Note that metal alkoxides would reduce volume during the hydrolysis and condensation reactions, and consequently the organic phase in the ceramer systems will become the main matrix except at very high composition ratios of metal alkoxide/organic such as the case over 80 wt % metal alkoxide in the creamer (for example, pure poly(Ti–O–Si precursor) in this work). Generally, the intensity of SAXS would increase with further polymerization forming a network structure, because densitification in the inorganic domains increases in the mean-square electron density variation. Densitification increased in the inorganic domains implies a more significant reaction within the system [20]. This organization generates a denser structure which is constituted by alternating inorganic and main organic linkages or bridges with a distance of several angstroms (Å). The theoretical mass density of this regime should depend on the curvature of dense structure [21]. Understanding of the above-mentioned theoretical background coupled with our XRD results can support our statement that the suppression of the inorganic phase by the amorphous polymer matrix. Because the inorganic composition (Ti–O–Si) covalently bonded on the organic molecular chains in the reactive precursors which polymerized with organic matrix enhancing further densitification in the Ti–O–Si frameworks, leading to shrinkage of the inorganic network during the densitification upon UV irradiation. This phenomenon is different from a physically mixed phase of the isolated crystalline particles blended in a polymer matrix in which the isolated crystalline particles still keep their original crystalline state. Moreover, this suppression is more significant with the further addition of reactive organic monomer (OPPEA) in the Ti–O–Si precursor, corresponding to the value of 0.58 nm. Pure poly(Ti–O–Si precursor) hybrid shows a crack film (Figure 5b), indicating that the shrinkage of molecular chains exists in the precursor hybrid during photopolymerization. Additionally, the shrinkage of inorganic-segment architectures is different from that of organic-segment architectures. The organic content is too low to completely connect the inorganic-segment architecture, leading to a crack state. Poly(Ti–O–Si precursor-co-OPPEA) hybrid demonstrates a compact film (Figure 5c), reflecting the addition of organic OPPEA is in compensation for the shrinkage of molecular chains in the Ti–O–Si segments during photopolymerization.

Because a large inorganic domain and refractive-index (RI) mismatch between the inorganic phase and the organic matrix, results in the significant light scattering of inorganic domains with the decline in transparency or opaque appearance of nanocomposites. The Rayleigh scattering formula can be employed to estimate the light scattering using transmittance (*T*) of the nanocomposites by randomly dispersed spherical particles with radius (*r*) and volume fraction (ϕ*_p_*) [14,22]:

(1) $$T=\frac{I}{I_{o}}=\text{exp}\left\{-\frac{32\pi^{4}\phi_{p}xr^{3}n_{m}^{4}}{\lambda^{4}}{\left[\frac{{\left(n_{p}/n_{m}\right)}^{2}-1}{{\left(n_{p}/n_{m}\right)}^{2}+2}\right]}^{2}\right\}$$

where *I* and *I*_o_ are the intensities of the transmitted and incident light respectively, λ is the wavelength of light, *x* is the optical path length, and *n_p_* and nm are the refractive indices of the inorganic particles and the organic matrix, respectively. To improve the transparency of nanocomposites minimizing the scattering loss at a given wavelength, the RI of the inorganic particles closely matches to that of the organic matrix, *n_p_* ≈ *n_m_*. Alternatively, the particle size needs to be significantly decreased below the visible light wavelength (200–800 nm). This is attributable that the large RI mismatch between the inorganic particles and the organic matrix can be compensated by ultra-fine particles to reduce losses by light scattering in designing high RI nanocomposites with high particle-loading level. Generally, the diameter of inorganic particles is below 100 nm. In this work, UV-vis spectra of Figure 6a indicates that poly(Ti–O–Si precursor-co-OPPEA) and poly(Ti–O–Si precursor) hybrid films at 633 nm have a transmittance of 91.1 and 25.2%, respectively. This implies that the hybrid polymer film consisted only of Ti–O–Si precursor readily results in light scattering and absorption, leading to a low transmittance. Conversely, the former film of poly(Ti–O–Si precursor-co-OPPEA) hybrid has lower light scattering and absorption with a higher transmittance.

Photopolymerization of organic-inorganic hybrid materials induced densification and molecular structural change. Therefore, the change of refractive index depends on the resultant film thicknesses. Recently, various organic–inorganic hybrid materials have been developed to combine organic and inorganic characteristics in an extraordinary hybrid material [23]. The hybrid materials can be photopolymerized by chain-extension or crosslinking of organic components using photo-initiators [24], whereas the organic–inorganic hybrid materials exhibit the changes in refractive index depending on film thickness after light illumination. The refractive index of poly(Ti–O–Si precursor-co-OPPEA) hybrid films (in Figure 7) increases with decreasing the film thickness in the heterogeneous hybrid films. This result is similar to thin films of SiO_2_-TiO_2_-PDMS composite material prepared by the sol-gel dip coating method after heating [25]. Upon UV irradiation, further densification enhances to extend the Ti–O–Si frameworks, leading to shrinkage of the inorganic network via the densitification. The Lorenz–Lorentz differential equation represents a refractive index change ∆*n* of a material [26]:

(2) $$\Delta n=\frac{\left(n^{2}-1\right)\left(n^{2}+2\right)}{6n^{2}}\left(\frac{\Delta \alpha }{\alpha }-\frac{\Delta \rho }{\rho }\right)$$

where Δα/α and Δρ/ρ are the relative changes in polarizability and density, respectively. From Equation (2), the refractive index change would be influenced by the two effects of the polarizability change and the density change. It is reasonable to assume that ρ change in density of a material is neglected in thin and thick films upon UV irradiation. Therefore, the contribution of density change cannot dominate the refractive index change in this hybrid material. The main effect for the refractive index change is the polarizability change (refer to Equation (2)). Because diphenyl groups on OPPEA molecules are relatively low polar as compared carbonyl groups and hence leads to a low refractive index of materials [27]. Thus, it is considered that the polarizability reduction in thicker films contributes dominantly to the decrease in refractive index on UV irradiation. On the other hand, the index of refraction is generally wavelength dependent in all transparent optical media. The variation of the refractive index with wavelength is dispersed. The dispersion of transparent optical materials is responsible for the splitting of light. This splitting is related to the actual motion of the electrons in the optical medium through which the light is travelling. In fact, the motion of electrons is more complicated in thicker hybrid film consisted of organic-inorganic heterogeneous two phases.

## 4. Materials and Methods

### 4.1. Chemicals

Titanium(IV) isopropoxide (TTIP, >98%) was purchased from Acros, Geel, Belgium. Methacryloxypropyltrimethoxysilane (MAPTMS) and 2-propenoic acid 2-([1,1′-biphenyl]-2-yloxy)ethyl ester (OPPEA) were from Aldrich, Milwaukee, WI, USA. Methyl alcohol (anhydrous, Macron Fine, Center Valley, PA, USA), nitric acid (69.0%–70.0%, J.T. Baker, Phillipsburg, NJ, USA), and 2-methyl-4′-(methylthio)-2-morpholinopropiophenone (Irgacure 907, Ciba, Basel, Switzerland) were of the highest commercially available purity and used as receive. Deionized distilled (DI) water prepared from a Milli-Q-RO water purification system (Millipore, Darmstadt, Germany) was purged with nitrogen for 30 min before use.

### 4.2. Synthesis of Organic-Inorganic Hybrid Sol-Gels

**Scheme 1:**
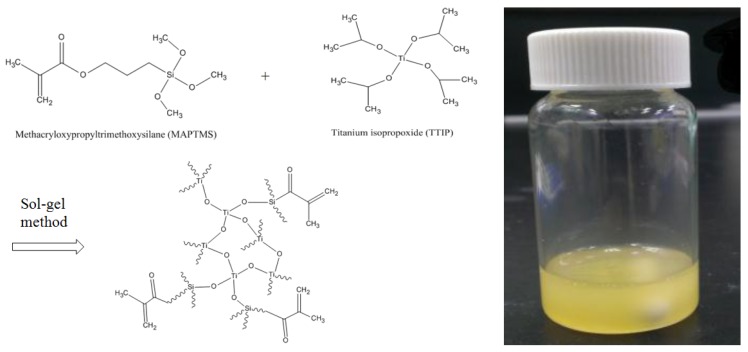
The preparation of Ti–O–Si-organic hybrid sol obtained from MAPTMS and TTIP Mixture appearance of sol, Irgacure 907, and OPPEA with a sol ratio of 4 wt %.

Ti–O–Si-organic precursors were synthesized by dissolving MAPTMS and TTIP in CH_3_OH (30 g) and then adding 1 g of HNO_3_ and 0.05 g of H_2_O; the total moles of MAPTMS and TTIP were kept at 0.042 with three TTIP/MAPTMS ratios of 9.3, 1.0, and 0.1, respectively. The mixture was stirred with purging nitrogen gas at 25 °C for 72 h. A milky white precipitate was collected by centrifugation at 8000 rpm. The preparation procedures of the inorganic-organic hybrid materials are shown in Scheme 1.

### 4.3. Preparation of the Organic-Inorganic Hybrid Coating

For photopolymerization, 7% (with respect to solid content) photoinitiator of Irgacure 907 (Ciba) was added to the sol or the mixture of the sol and OPPEA (control the ratio of sol/OPPEA = 4 wt %). Then the above solutions were spin-coated on the glass substrate with an area of 2.5 × 2.5 cm^2^ at 2000 rpm for 60 s. The film was exposed in a MJB3mask aligner with a UV 400 filter for 10 min. The power level was 8 mW/cm^2^ measured at 365 nm.

### 4.4. Measurements

The size distribution of sol particles was measured by a dynamic light scatter (Brookhaven, Holtsville, NY, USA, 90 plus). Infrared spectra were recorded on a PerkinElmer RXI FT-IR spectrometer (Waltham, MA, USA). Absorption spectra were measured with a Perkin-Elmer Lambda 25 UV-visible spectrophotometer. The microstructure of the sample was investigated by scanning electron microscopy (SEM, Hitachi, Tokyo, Japan, S-4800). Material thermal stability was measured by the thermogravimetry analysis (TGA, SDT-Q600, TA Instruments, New Castle, DE, USA). The refractive index of the optical films was measured by using the N & K analyzer (1280, n & k Technology, San Jose, CA, USA). The reflection and transmission spectroscopy were first measured and the data were calculated by using the Forouhi-Bloomer model to test the goodness of the fit. This process generates the values of refractive index (*n*) and extinction coefficient (*k*) which provide a basis of data to produce a new reflection curve through calculation. The curve fitting was repeated until this new reflection curve closes to the measured curve with the goodness of fit greater than 0.99. The obtained refractive index and extinction coefficient are reliable after the calculation and curve fitting. In the fitting process, the film thickness can simultaneously be obtained.

## 5. Conclusions

Organic-inorganic hybrid sols (Ti–O–Si precursor) were synthesized by the sol-gel method at low addition of water, and were then blended with 2-phenylphenoxyethyl acrylate (OPPEA) to perform photopolymerization by UV irradiation to prepare highly refractive hybrid optical films. The hybrid film of poly(Ti–O–Si precursor-co-OPPEA) exhibited a high transparency of ~93.7% with a high refractive index (*n*) of 1.83 and thickness of 2.59 μm at the absorption wavelength of 633 nm. In this work, the refractive index of the polymer encapsulation materials was raised to 1.83, giving the efficiency of light emission the opportunity to be promoted about 40% in the LED application. The high-refractive-index materials can also be excellent candidates in the applications of optical devices, optical storage materials, anti-reflection films, and solar cells.
